# Can Iron Play a Crucial Role in Maintaining Cardiovascular Health in the 21st Century?

**DOI:** 10.3390/ijerph191911990

**Published:** 2022-09-22

**Authors:** Michał Szklarz, Katarzyna Gontarz-Nowak, Wojciech Matuszewski, Elżbieta Bandurska-Stankiewicz

**Affiliations:** Clinic of Endocrinology, Diabetology and Internal Medicine, School of Medicine, Collegium Medicum, University of Warmia and Mazury, 10-957 Olsztyn, Poland

**Keywords:** iron, heart failure, diabetes, obesity, COVID-19

## Abstract

In the 21st century the heart is facing more and more challenges so it should be brave and iron to meet these challenges. We are living in the era of the COVID-19 pandemic, population aging, prevalent obesity, diabetes and autoimmune diseases, environmental pollution, mass migrations and new potential pandemic threats. In our article we showed sophisticated and complex regulations of iron metabolism. We discussed the impact of iron metabolism on heart diseases, treatment of heart failure, diabetes and obesity. We faced the problems of constant stress, climate change, environmental pollution, migrations and epidemics and showed that iron is really essential for heart metabolism in the 21st century.

## 1. Introduction

Can iron play a crucial role in maintaining cardiovascular health in the 21st century? We are going to prove that the headline question is merely rhetorical. In the 21st century the heart is facing more and more challenges so it should be brave and iron to meet these challenges. In the era of the COVID-19 pandemic, population aging, prevalent obesity, diabetes and autoimmune diseases, environmental pollution, mass migrations and new potential pandemic threats, iron is really essential for cardiovascular health and heart metabolism. Is it not ironic that deficiency of this microelement is among the most prevalent nutritional deficiencies? Iron deficiency (ID) is estimated to affect as many as 2 billion people all over the world [1], mostly pregnant women and children. In large population studies ID was reported in 41.7% of children aged less than 5 years and 40.1% of pregnant women [2]. In the United States of America ID is diagnosed in 11% of children aged between 6 months and 5 years and in 18% of pregnant women [3]. It is the underlying cause of anaemia in 42% and 50% of anaemia cases in children and women, respectively. ID is also among the five most frequent causes of disability in women from 35 countries [4]. The aim of the study is to assess the impact of the iron deficiency on human cardiovascular health in the perspective of 21st century challenges.

## 2. Material and Methods

The study presents an analysis of data in the currently available literature. We examined electronic databes, including: MEDLINE and Pubmed. The search terms we used included: “iron deficiency”; “iron deficiency anemia”; “iron and heart failure”; “iron and heart failure treatment”; “iron and heart metabolism”; “iron and obesity”; “iron and diabetes”; “iron and immunology”; “iron metabolism in COVID-19”; “iron metabolism and enviromental pollution” and “iron and stress”.

## 3. Iron Metabolism in the Finer Details

Ferrum is an essential and vital element, but can also be toxic. Therefore, sophisticated and complex regulations are needed to avoid both shortage and excess of this element [5]. Sufficient iron delivery is essential as all body cells use iron as a cofactor in key biochemical reactions, oxygen transportation, metabolism and DNA synthesis [6]. Two percent of human genes encode iron-binding proteins and 6.5% of all enzymes are directly dependent on iron [7]. ID can cause microenvironmental and immune changes in the human body. It can potentiate DNA damages and genome instability. It is one of the main causes underlying the imbalance between antioxidant enzymes and enzymes involved in DNA damage and repair. It may affect biogenesis and expression of microRNA (molecules able to modify DNA). ID impairs oxidative phosphorylation and causes dysfunction of the respiratory complex enzymes (succinate-ubiquinone reductase and NADH-coenzyme Q oxidoreductase) [8,9], it can also reduce the number and volume of mitochondria and decrease mitochondrial cristae density [9].

Iron homeostasis is regulated by balancing its absorption, transport, influx into cells, storage, incorporation into proteins and transport from one cell to another [5]. The average amount of iron in the human body is 9.7 mg/kg body weight (b.w.) in men, 5.7 mg/kg b.w. in premenopausal women and 7.8 mg/kg b.w. in postmenopausal women [10]. The great majority of iron is bound in haemoglobin (approx. 2.1 g) within red blood cells (RBCs) and is used for oxygen transport—600 mg is found in macrophages, 300 mg in myoglobin, while liver iron stores amount approximately 1 g (which corresponds to approximately 60% of the total iron stores in the body) [11]. The remaining 40% is stored in muscles and reticuloendothelial tissues. Usually 95% of the iron stored in the liver is bound in ferritin and 5% in hemosiderin. An amount of 0.1% of the total iron is circulating in the serum in form of transferrin, and 20–25 mg of iron daily is used for red blood cell production and cellular metabolism [12].

Inorganic iron is absorbed at the apical membrane of duodenal enterocytes and is reduced from ferric iron (Fe^3+^) to ferrous iron (Fe^2+^) by duodenal cytochrome B (DTCYCB). Fe^2+^ is then transported across the cellular membrane by the divalent metal transporter (DMT1). Having passed into the cytosol, the elemental iron can exit the cell across the basolateral membrane with the help of ferroportin (which is a ion transporter). The ferrous iron can be oxidized back to the ferric form by hephaestin. Fe^3+^ is bound to transferrin and further distributed to all body cells in the form of transferrin-iron complex (holo-transferrin-holo-TF). Haem iron is absorbed by the HCP1 (haem/folate transporter-1) and can exit the cell with the help of the FLVCR-haem transporter at the basal membrane or undergo oxidation by haem oxygenase and return to the cytosol [13].

Red blood cells, which survive 120 days on average, contain 80% of the functional iron in the human body. They use iron, binding it to haem and iron-sulphur clusters (ICS). Synthesis of the tetrapyrrole rings of haem from the 5-aminolevulinic acid (ALA) precursor occurs via the eight-step enzymatic pathway. ALA is formed as a result of condensation of succinyl-CoA and glycine, which is catalysed by ALA synthase (ALA1 and red-blood-cell-specific ALA2). ALA precursor is exported to the cytosol and converted into porphobilinogen, hydroxymethylbilane, uroporphyrinogen III and coproporphyrinogen III, with the last one being oxidised into protoporphyrinogen IX, which is then transported into the mitochondria, where oxidation to protoporphyrin IX occurs. The last step in this process is binding Fe^2+^ into protoporphyrin IX. The resultant haem is exported to the cytosol and incorporated into the haemoproteins. Haem and haem metabolites are transported across the cellular membranes with the help of ABC transporters and SLC25a39 [14]. A rate-limiting factor in the synthesis of haem in red blood cells is iron supply, which regulates the key step of the biosynthesis by changing the stability of mRNA for the synthesis of ALA [5]. To explain the transfer of iron to the RBC mitochondria, the ‘kiss and run’ hypothesis has been proposed, which suggests that iron is imported to the mitochondria via the transient contact of transferrin-containing endosomes with mitochondria [15]. In other types of cells iron is unbound from transferrin and transported in the cytosol. An important role in the transport of iron is played by the conserved cytosolic glutaredoxins Grx3 and Grx4 [16]. For iron influx into the mitochondria the SLC-transporter (mitoferrin-SLC25a37) is required, which is localized in the mitochondrial inner membrane [17]. In case of ID, red blood cells deliver iron via ferroportin, which makes them a kind of buffer when this element is deficient in the serum, and protects them against oxidative stress. This breakthrough discovery suggests that microcytic anaemia is not necessarily the last step in the ID states, because according to the most recent evidence red blood cells are able to transfer iron to other tissues [18]. Iron recycling involves the ageing RBC destruction in the reticuloplasmatic system [19]. In a man with a body weight of 70 kg, approximately 35 mg of iron daily is returned to the serum—which makes 0.66% of the total body iron [20]. The CD163 receptor, found on the monocytes, macrophages, microglia and neurons, plays an extremely important role in the iron recycling in the states of bleeding, neuronal damage and iron overload. Excess iron is removed from cells by ferroportin or haem secretion by FLVCR [19]. Cells can also dispose of excess iron by storing it in ferritin, of which two subunits have been identified: H and L. Binding iron to ferritin to produce holo-ferritin requires the activity of ferro-peroxidase, which is present in H-ferritin, while L-ferritin provides the nucleation centre. The subunit H is found mostly in the heart whereas the subunit L predominates in the liver. Intracellular iron is also stored in the form of hemosiderin. The iron-storage function of ferritin is vital to maintain life, the ablation of the gene encoding H-ferritin has been found to be embryolethal [21], and dysfunction of the gene results in liver damage. L-ferritin mutations lead to neuroferritinopathy, a disorder in which the redox-active form of iron accumulates in the brain.

The biological role of iron depends on its chemistry, as iron can take three different forms: Fe^2+^ (*ferrous*), Fe^3+^ (*ferric*) and Fe^4+^ (*ferryl*). During its conversion from one degree of oxidation to another the iron transports electrons and binds to ligands by virtue of its unoccupied d orbitals. The preferred ligands for iron are as follows: nitric oxide, oxygen and sulphur. The electron spin and redox potential of iron (from +1000 mV for some haem proteins, to −550 mV for bacterial ferredoxins) can change to adjust to specific ligands. The ability of iron to change its oxidative potential, redox potential and electron spin state is reflected in the enormously large number of chemical reactions [22]. Groups of proteins that require the presence of iron include haemoglobin and myoglobin, iron-sulphur clusters involved in electron transport and energy metabolism, haem-containing enzymes involved in electron transport in cytochromes, as well as other iron-containing enzymes [5].

Oxygen delivery from the external environment up to the mitochondria is one of the main functions of iron. Iron is bound into the porphyrin ring either as a part of the prosthetic group of haemoglobin or as a tissue oxygen delivery facilitator bound into myoglobin. Each Hb subunit contains one prosthetic group (Fe-PP-IX), in which Fe^2+^ is reversibly bound to oxygen [5]. The cytochrome family contains haem in its active site, with an iron atom in the porphyrin ring able to transform from the Fe^2+^ form to the Fe^3+^ form by accepting an electron. Iron-sulphur clusters serve as electron transporters, binding an iron atom to two or four sulphur atoms and sulphur side chains. Iron deficiency sensitive enzymes are as follows: pyruvate-lactate oxidase (activity reduction by 30 ± 3%) succinate oxidase (29 ± 5%) and cytochrome oxidase (55 ± 9%) (muscle mitochondrial volume is reduced by 72 ± 8% while muscle cytochrome oxidase activity is reduced by 48 ± 7%) [5].

Hepcidin is the central regulator of iron homeostasis. It is produced in the liver, macrophages, heart, adipocytes and kidneys. By binding to ferroportin, hepcidin controls inorganic ion absorption, which results in a decrease in iron transfer across the basolateral enterocyte membrane [13]. In states of iron deficiency or increased iron requirement, hepcidin suppression can increase the absorption and recycling of iron. ID results in increased levels of apotransferrin (iron-free protein), which is unable to dissociate HFE (human homeostatic iron regulator protein) from transferrin receptor 1 (TFR1). A lack of HFE in the plasma makes the iron sensing complex inactive and as a result hepcidin is not produced [13]. Iron-sensing complex formation is potentiated by higher HFE levels and contains the following proteins: transferrin receptor 2 (TFR2), HFE, hemojuvelin and bone morphogenetic protein receptor 2 (BMPR). The complex activates the SMAD pathway, which upregulates hepcidin expression [12]. Inflammatory states are accompanied by increased hepcidin synthesis and decreased ferroportin transcription, which results in lower iron availability and its functional deficiency, which sometimes may co-exist with its systemic deficiency owing to depleted body iron stores [23]. Chronic inflammatory conditions can increase hepcidin levels and decrease iron absorption—as reported in a study from the Ivory Coast, where, in women with asymptomatic plasmodium falciparum infection iron absorption was considerably increased after antimalarial treatment [24]. In another study from Gambia, a chronic inflammatory state, even mild, in children aged 6–23 months, resulted in increased hepcidin levels and impaired absorption of iron [25]. Hepcidin levels are reduced by increased testosterone, oestrogen, growth hormone, and vitamin D. Low hepcidin levels indicate an increased iron requirement, predict iron responsiveness, and may help find the optimal route for iron supplementation [26]. Hepcidin measurements are likely to become the most sensitive parameter for the assessment of iron metabolism balance on a tissue level. Murine studies have shown that mice devoid of hepcidin had considerably shorter lifespan, decreased ejection fraction and cardiac hypotrophy. Although their total body iron was quite good, a marked reduction in their respiratory complex activity was observed [27].

## 4. Definition and Importance of Iron Deficiency

The WHO recommends 60 mg elemental iron daily. The recommended daily allowance (RDA) for iron is the highest for infants aged 7–12 months, which is 11 mg- the iron RDA for premenopausal women is 18 mg, that for pregnant women is 27 mg- and that for men is 8 mg. The usual diet provides 1–2 mg of iron daily, whereas iron bioavailability from dietary supplements and medicinal products is as low as 10% [28]. It is even lower in developing countries owing to the previously mentioned inflammatory conditions that result in hepcidin upregulation and iron malabsorption [29].

The gold standard for the diagnosis of ID is the absence of stainable iron in a bone marrow aspirate. However in patients with functional ID stainable bone marrow iron is detected until their body iron stores are depleted. In a study of healthy women ferritin levels < 15 mcg/L predicted bone marrow iron depletion with 75% sensitivity, while shifting the cut-off value up to 30 mcg/L increased the sensitivity to 93% [30]. In inflammatory conditions the cut-off value is proposed to be 30 mcg/L in children and 70 mcg/L in adults [31]. In patients with chronic kidney disease this threshold is moved up to 100 mcg/L, in dialysis patients to 200 mcg/L and in heart failure patients to 100 mcg/L or between 100 and 300 mcg/L if transferrin saturation (TSAT) is <20% [32]. The most recent research shows that the cut-off value in autoimmune thyroid disease (AITD) should be 70 mcg/L [33]. Additionally to hepcidin, another marker useful for predicting functional iron deficiency is sTFR, which defines the amount of iron available for tissues. The sTFR/log ferritin ratio is highly sensitive in estimating the bone marrow iron store [34].

With each heart muscle contraction 60–70 mL of blood is ejected from the heart into the aorta, and it takes 60 s to pump 5 mL of blood through the heart. There are on average 50,000 drops of blood circulating in the human body. Every single drop (0.1 mL) of blood counts, containing approximately 0.04–0.05 mg of iron. Severe menstrual bleedings occur in almost 20% of women [35] whereas in men and postmenopausal women the most prevalent cause of blood loss is gastrointestinal bleeding. In the group of ID patients referred for endoscopic procedures a potential site of bleeding was found in 62% of patients (colorectal cancer in 11%, gastric ulcers in 19%) [36]. Even in the male population under the age of 50 colorectal cancer is diagnosed in 0.8% of patients with iron deficiency [37]. The risk of gastrointestinal cancer at 2 years after making the ID diagnosis was 6% in men and 1% in postmenopausal women versus 0.2% in subjects without ID [38]. Moreover the epidemics of using proton pump inhibitors (PPIs) can increase the risk of ID and the relationship is dose-dependent [39]. The risk of ID is also increased by the high prevalence of Helicobacter pylori infection (which affects 4.4 billion people worldwide) while H. pylori eradication has been shown to improve iron supply [40]. Considering patients with ID we must remember celiac disease, as this enteropathy is finally confirmed in 1 out of 31 patients undergoing diagnostic procedures because of iron deficiency.

## 5. Iron Deficiency and Heart Failure—Litmus Papers of Inequality

Iron supply highly depends on socioeconomic status. This fact is clearly visible in population studies, which indicate that ID affects 4–18% of the population of the USA and Northern and Western Europe versus 9–50% of the population of Eastern Europe, 64% of Asia and 62% of Latin America [12,13]. It is noteworthy that iron deficiency and concomitant iodine deficiency still affects 30% of children in Western African countries [41]. Furthermore, iron deficiency anaemia affects even every fourth child in developing countries [41] and may result from a monotonous diet of low nutritional value, endemic diseases, parasitic infections, and malaria. In the era of mass migrations caused by economic inequalities, war, or climate changes, particular attention should be given to migrants and refugees who are most severely affected by ID [42] with its all consequences for health and assimilation prospects. According to the United Nations Refugee Agency (UNHCR), 65.6 million people have been forcibly displaced worldwide [43]. A refugee’s health profile is markedly different from that of the population in the country of asylum [44].

The population of refugees is very vulnerable to malnutrition, infectious and chronic diseases. These last ones can lead to absolute and/or functional iron deficiency [44].

In the study of Stellinga—Boelen et al. carried on 122 asylum seekers’ children in the Netherlands iron deficiency was observed in 20% of the research group [45]. In populations of African refugee camps prevelence of ID was high, ranging from 23 to 75% [46]. In another analysis of 1131 new paediatric refugees in Australia 12.3% had ID [47]. Socioeconomic deprivation is not only a strong and independent risk factor of ID, but also of chronic heart diseases, such as arterial hypertension and heart failure [47]. Migrants are also more likely to develop chronic coronary syndrome [48]. Furthermore, People with low-income and a lower education status are less likely to receive effective treatment of heart fauilure [49,50]. In Swedish research, a lower socioeconomic status was associated with higher risk of HF hospitalisation/mortality, as well as overall cardiovascular and non-cardiovascular events [51]. The precise mechanisms accounting for this risk still remain unclear. The interaction between socioeconomic status and HF is complex–however, iron deficiency may possibly be one of the factors involved.

## 6. Iron and the Heart

### 6.1. Iron in Heart Metabolism

In the aging population there is a growing prevalence of chronic heart failure (HF). HF is accompanied by systemic and functional iron deficiency [52]. The underlying causes of iron deficiency in HF include reduced protein uptake, fluid overload, antiplatelet drug use, increased hepcidin levels and intestinal wall oedema [53,54]. Moreover a typical feature of heart failure is chronic inflammation, which results in increased sympathetic tone and reduced erythropoetin (EPO) levels [55]. In cardiomyocytes iron plays a key role as a component of mitochondrial enzymes and, owing to the oxygen carrying function of myoglobin–ID leads to dilated cardiomyopathy in rats, with impaired mitochondrial structure, abnormal sarcomere assembly, and changes in Rhoa kinase expression. Morphologic examination showed that the major/minor ventricular diameters and ventricular volume were significantly enhanced in ID rats [56]. Heart failure is associated with decreased activity of iron regulatory proteins (IRP) and reduced amount of tissue iron. Cardiac specific deletion of IRP-1 and IRP-2 resulted in impaired contractility in the dobutamine stress test while iron supplementation was able to restore normal cardiac function [57]. A study of human cardiomyocytes depleted of iron by incubation with deferoxamine has shown a decrease in the activity of enzymes with iron-sulphur clusters in complexes I, II, and III. A reduction in cellular ATP by 74% and reduction in contractile force by 43% were observed. The maximum velocities during both systole and diastole were reduced by 64% and 85%, respectively [58]. ID considerably contributes to the destruction of the cytoskeleton of vascular smooth muscle cells (VSMCs) in cardiomyocytes [59]. Changes in the expression of mitochondrial protein genes involved in cellular iron metabolism (Ftmt, Mtfrn 1/2, frataksyna, Glrx5, ABCB6, ABCB7, ABCB10) may lead to the development of cardiovascular disease [31].

### 6.2. Anemia in Heart Failure

Heart failure affects 26 million people globally [60]. It is classified into two major subtypes: HFrEF (heart failure with reduced ejection fraction) and HFpEF (heart failure with preserved ejection fraction). HF is diagnosed in 1–2% of people in Western countries with prevalence reaching 10% in people aged more than 70 years [61], which makes it the most frequent cause of hospitalizations all over the world [62]. The risk of HF at the age of 55 is 33% for men and 28% for women [63].

According to some studies anaemia affects as much as 30% of patients with stable HF and 50% of hospitalized patients [64]. In the Val-HeFT study anemia developed in 16.9% of patients with HF, and 25% of patients with the biggest decrease in Hb had a 1.6-fold increase in mortality [65]. In the SOLVD study anemia developed in 9.6% of HF patients and was associated with a 2-fold increase in the risk of death [66]. The pathogenesis of anemia in HF is multifactoral. Several causes of anemia in HF have been iddentified: renal impairment, inflammatory state, medications and hemodilution [67]. These factors can lead to the development of chronic disease-associated anemia [68]. It is noteworthy that vitaminum B12 and folate deficiencies are infrequent-however, ID is extremely common [69]. Patients with HF and concomitant anaemia suffer from more severe oedema, require higher doses of diuretics, are more likely to develop chronic kidney disease and diabetes, as well as have a lower quality of life (QoL) and lower exercise capacity. Moreover, anaemia is associated with higher severity of HF and a lower outcome of treatment [64,70]. Very severe anemia can cause HfpEF that results in an increase in Hb [71], leading to an increase in myocardial workload to compensate for the reduced tissue oxygen delivery. Some studies showed that erythropoetin (EPO) improves exercise capacity and NYHA class and reduces hospitalization [72]. Moreover, EPO can improve LVEF, right ventricular ejection fraction left ventricular function [73], as well as supress proinflammatiory citokines (TNF-alfa, IL-6) with an increase in antiinflammatory IL-10 [74]. Van Veldhuisen et al. in their study carried out on 165 patients with HF observed that darbepoetin alfa increased Hb and improved the Kansas City Cardomyopathy Questionnaire score [75]. In STAMINA-HF (Study of Anemia in Heart Failure) carried out on 319 patients, the group-in which the Hb increase was at least 2 g/dL-showed the greatest improvement in exercise duration [76]. In study of Palazzuoli et al. in patients with HF, an improvement in NYHA and V02 after EPO was observed [77].

### 6.3. Observational Studies of Iron Deficiency in Heart Failure

A large body of evidence has confirmed an increased prevalence of ID in heart failure patients. ID is found in nearly 50% of patients with HF [32]. ID becomes more prevalent in patients with a higher NYHA class, increases the mortality and morbidity of patients with HFrEF, and impairs exercise tolerance [69]. In a German study of 1198 heart failure patients, ID was found in as many as 42.5% of the patients [78]. In another study of 4456 HF patients anaemia was diagnosed in 27.8% of study subjects (mild anaemia in 14.4%, moderate anaemia in 7.9% and severe anaemia in 5.4%). The highest prevalence of anaemia was observed in the group of patients with left ventricular systolic dysfunction (LVSD) (33.3%). Iron levels lower than 45 mcg/L were found in 14% of patients, and lower than 67 mcg/L in 36.6% of patients. Ferritin levels < 30 mcg/L were seen in 10.7% of patients and lower than 100 mcg/L in 43.8% [79]. In a study of 165 patients suffering from acute heart failure, those with low hepcidin and high sTfR were more likely to have peripheral oedema and increased nt-proBNP and uric acid levels. During 12 month follow-up 20% of the study patients died including 15% of those with isolated increased sTfR, and 7 of those with isolated decreased hepcidin [80]. ID in cardiomyocytes as expressed by high a T2 value in cardiac magnetic resonance (CMR) imaging is associated with nonischaemic heart failure and impaired left ventricular function. An high T2 CMR value may predict the risk of major adverse cardiovascular event (MACE) in patients with nonischaemic HF [74]. In a study of 903 patients a correlation was found between ID and right ventricular dysfunction in acute heart failure. Lower transferrin saturated with iron (TSAT) and ferritin were independently correlated with lower tricuspidal annular plane systolic excursion (TAPSE) [81]. Another study of 111 patients has shown that the risk of myocardial infarction and cardiovascular morbidity was increased in ID patients by 50% at 4 years [82].

### 6.4. Interventional Studies of Iron Deficiency in Heart Failure

Bolger et al. ([83]; Table 1) treated their HF and anaemia patients with intravenous iron administered in the dose of 1 g daily for 12 days and reported an improvement in Hb level and alleviation of HF symptoms. In a study by Toblli et al. ([84]; Table 1), which included 40 patients with NYHA II–III and LVEF < 35% administered 200 mg of iron for 5 weeks, an improvement in nt-proBNP, NYHA, and Minnesota Living with Heart Failure Quality of Life Score was observed along with a reduced requirement for diuretics, improvement in the 6 Minute Walk Test (6MWT) and left ventricular ejection fraction (LVEF) as well a decrease in C-reactive protein and creatinine levels. In the group of patients with congenital heart disease with pulmonary arterial hypertension (CHD-PAH) 39% of patients had iron deficiency; these patients had higher nt-proBNP levels and a shorter walking distance in the 6MWT [85]. In another interventional study (FAIR-HF study) the therapy with ferric carboxymaltose administered for 24 weeks in patients with HF and ID resulted in a considerable improvement in QoL, 6MWT distance increased from 274 ± 6 to 313 ± 7 m, and the mean Kansas City Cardiomyopathy Questionnaire (KCCQ) score improved from 52 ± 1 to 66 ± 1, with the first benefits seen as early as at 4 weeks of treatment ([86]; Table 1). Okonko in 2008 ([87]; Table 1) reported on 35 HF patients administered 200 mg of iron weekly until their ferritin increased up to 500 ng/mL and noticed an improvement in NYHA class, peak VO2 and QoL. Beck da Silva et al. (IRON-5 study) ([80]; Table 1) reported 18 female patients, who were administered an iron (III) hydroxide—sucrose complex in the dose of 200 mg for 5 weeks, where considerable increase in oxygen consumption was observed as their peak VO2 and QoL improved. In the CONFIRM-HF study ([88]; Table 1) of 304 patients randomized to receive 500 mg or 1000 mg of ferric caroboxymaltose a more significant improvement in NYHA class, 6MWT distance and QoL was observed in the group administered the 1000 mg dose. The study by van Veldhuisen from 2017 ([89]; Table 1), which reported 172 patients administered 1000 mg of ferric carboxymaltose has shown an improvement in NYHA class, peak VO2, and QoL. ID can have negative effects on the lungs in rats–including increased expression of hypoxia-inducible factor (HIF)-1alpha, HIF2-alpa, nuclear factors of activated T-cells, survivin, transcription factors and signal transduction factors, which promotes smooth muscle cell proliferation and contributes to apoptosis resistance. Moreover reduced activity of the mitochondrial complex I and hyperpolarization of the mitochondrial membranes is observed, which may lead to vascular hemodynamic changes and subsequent pulmonary hypertension [90]. A study of 53 male patients suffering from stable heart failure with reduced ejection fraction (HFrEF), with no concomitant pulmonary disease, in NYHA class I–III, with EF < 40%, investigated oxygen metabolism in skeletal muscles. A low ferritin level was associated with inspiratory muscle weakness, independently of the total muscle mass [91]. In the group of patients with ID, iron supplementation improved skeletal muscle function in HF patients, and phosphocreatine recovery half time (PCr T1/2) reached better values within 2 weeks, suggesting cardiomyocyte mitochondrial function improvement ([92]; Table 1). ID can also lead to a hypercoagulable state, which is reversible upon iron replacement therapy [93].

The European Society of Cardiology recommends that ferric carboxymaltose should be administered to all patients with symptomatic heart failure and reduced ferritin.

### 6.5. Iron and Treatment of Heart Failure

The last decade of the 21st century was a very exciting time for cardiologists and diabetologists as the major scientific advances and breakthrough discoveries resulted in a real revolution in the treatment of heart failure and diabetes. Agents from the class of sodium-glucose co-transporter 2 (SGLT2) inhibitors exert a range of positive effects that result in the cardiovascular risk reduction [94] and reduction in HF hospitalizations and cardiovascular events [94]. In addition to their glucose-lowering effect, they also exert a natriuretic effect and cause a shift in energy metabolism with the promotion of ketone bodies, which are considered a better “fuel” for the heart. Ketone bodies are selectively taken up by cardiomyocytes instead of glucose and fatty acids. After 3–4 weeks of empagliflozin treatment a 3–4-fold increase in serum ketones is observed [95]. Ketones are used with more effective myocardial oxygen consumption, which may improve heart function and reduce the risk of heart failure. Noteworthy, in the iron deficiency states ketogenesis is impaired with a subsequent reduction in citrate synthase and succinate dehydrogenase activities and impaired production of free fatty acids (FFAs) and ketone bodies [96,97]; therefore iron deficiency can indirectly diminish the effects of the flozin therapy. Moreover, SGLT2 inhibitors can increase cardiomyocyte oxygenation as they increase haematocrit, reduce intraglomerular pressure, affect sodium-hydrogen exchange in the heart and in the kidneys and improve left ventricular contractility [60]. Inflammation and overactivity of the renin-angiotensin-aldosterone system (RAAS) and sympathetic nervous system inhibit iron cellular uptake by blocking transferrin receptor [98]. Flozins reduce inflammation in the heart and in the kidneys [99]. There is a body of evidence to show that patients treated with SGLT2 inhibitors have lower hsCRP levels [100,101]. Dapagliflozin can reduce the cardiac and renal levels of norepinephrine in states of sympathetic hyperactivity [102]. This mechanism can explain why dapagliflozin administration can result in circulating blood volume and pressure reduction with no effect on heart rate [103]. Therefore SGLT2 inhibitors have been speculated to improve transferrin receptor function and tissue iron uptake. Flozins are likely to enhance iron absorption because they selectively reduce the interstitial fluid volume while their effect on the plasma fluid volume is minimal [104,105]. They decrease intestinal oedema, increase erythropoietin levels, and increase haematocrit [106]. The increase in EPO levels seen with SGLT2 inhibitors may be responsible for their organ protective properties and pleiotropic effects, which results in improved cardiac muscle function, angiogenesis, cell proliferation, and reduction in inflammation [98]. The EMPA-HEART CardioLink 6 study has shown an increase in EPO levels in type 2 diabetes mellitus (T2DM) patients with chronic coronary syndrome administered empagliflozin [98]. In a 24-week observational study, dapagliflozin at 5 mg daily has been shown to increase ferritin levels in T2DM patients with non-alcoholic steatohepatitis (NASH) [107]. Canagliflozin at 100 mg daily also increased ferritin in T2DM patients with non-alcoholic fatty liver disease (NAFLD) [108]. The CREDENCE study involving 4401 patients has confirmed that canagliflozin-treated patients had a lower risk of anaemia and were less likely to receive iron supplements and erythropoiesis stimulating factors [109]. SGLT2 inhibitors lead to blood thickening owing to their natriuretic effect [110,111]; however only slight increases were observed in albumin and total protein, so dilution cannot be the only mechanism to explain the increase in haematocrit. EPO rises with subsequent increases in RBC mass and reticulocytes seen at 2–4 weeks of SGLT2 inhibitor therapy [110,112]. SGLT2 inhibitors lead to improvements in the hypoxic microenvironment in the proximal tubule [113]. They decrease the ATP-workload in the proximal tubule, which can reverse the conversion of myofibroblasts to EPO-producing fibroblasts. Moreover, SGLT2 inhibitors can inhibit hepcidin, which may lead to increased iron bioavailability and utilization [114]. Dapagliflozin has been shown to reduce hepcidin levels by increasing the hepcidin inhibitor—erythroferrone. It can also increase transferrin levels and transferrin receptor 1 and 2 expression in mononuclear cells (MNCs), and decrease HIF1 expression by increasing the expression of its inhibitor propyl hydroxylase-2 [114]. SGLT2 inhibitors also lead to an increase in copeptin—a surrogate marker for vasopressin, which stimulates EPO [115].

Elevated levels of catecholamines and aldosterone in heart failure down-regulate Tfr1 and Tfr2 receptors, which results in reduced iron uptake by cardiomyocytes. Beta-blocker or mineralocorticoid receptor antagonist (MRA) therapy, by normalizing TfR1 and TfR2 expression, is able to improve cardiomyocyte iron uptake [116]. Spironolactone has been shown to suppress hepcidin in mice [117], with the potential to increase iron availability.

A weak correlation was found between angiotensin-converting enzyme inhibitors (ACEIs) and iron deficiency and anaemia [118]. As A2 stimulates erythroid cells, a decrease in A2 level can decrease erythroid cell proliferation. ACEIs also inhibit IGF-1 secretion, which stimulates bone marrow progenitor cells [119]. During ACEI therapy an increase in erythropoietin inhibitor, N-acetyl-seryl-aspartyl-lysine-proline, is observed [120]. ACEIs could modify the course of Fenton reaction, which is of key importance for iron chemical function, and could form different iron complexes depending on the order of addition of reagent. These complexes induced the generation of oxidizing intermediates [121]. ACEIs stimulate the production of nitric oxide (NO)—a substance with a proinflammatory effect in the bronchial endothelial cells. Iron, being an NO synthase inhibitor, can reduce the frequency of dry cough during ACEI therapy [122]. Beta-blockers, thanks to their anti-inflammatory properties, can improve iron metabolism balance in HF [123]. Calcium channel blockers (CCBs) are able to inhibit erythropoiesis and administration of these agents may be associated with lower haemoglobin values. Calcium is vital for normal differentiation and maturation of erythroid cell precursors. EPO, stimulating the precursors, can lead to increased intracellular calcium and AMP [124]. With CCB therapy precursor cells become resistant to EPO because cellular calcium uptake is inhibited [125]. Moreover, patients administered calcium channel blockers have been shown to have a higher risk of gastrointestinal bleeding [126], as well lower ferritin levels and lower DMT-1 expression [127].

## 7. Iron and Muscle Performance

Normal muscle function is necessary to maintain physical fitness at any age. A sedentary lifestyle is a real epidemic of the 21st century. In modern busy societies, finding time for physical exercise is nearly impossible. Between 20 and 50% of HF patients develop sarcopenia, which is a marker of a poor outcome in patients with ID [94]. What is important is that, ID can also a reduce patient’s ability to recover after myocardial infarction and stroke [128]. Blood oxygen transported with haemoglobin and cardiac output are the key factors that determine muscle function. Baynes and Bothwell in 1990 [129] indicated that ID is frequently accompanied by lethargy, apathy and reduced physical activity. Dallman et al. reported a decrease in transported oxygen and muscle oxidative capacity [130]. Moreover there is lower content of the mitochondrial iron-sulphur clusters [131], cytochrome enzymes [132], and total mitochondrial oxidative capacity in the muscles [133]. In ID, the ability to exercise is impaired by approximately 80% versus the control group [134]. Iron treatment resulted in an improvement in these disorders as early as within 4 days, but mitochondrial oxidative capacity, as expressed by the consumption of substrates such as pyruvates, lactates and succinates did not improve. In the study by Thomson from 1993 [135], ID contributed to more rapid phosphocreatine decrease and lower pH, as well as impaired post-exercise regeneration of phosphocreatine and inorganic phosphates. In the study by Dallman et al. anaemia was found to reduce oxygen supply to the muscles, while reduced tissue iron diminished the effectives of oxygen metabolism [130]. Lower enzymatic activity in the muscles and liver results in increased muscle lactate production [133]. Further studies confirmed an increase in lactate dehydrogenase activity in the muscles in the case of ID, which is associated with muscle adaptation to the maximum use of energy from anaerobic metabolism [136]. Gluconeogenesis markers in ID are increased in order to ensure appropriate glucose supply to tissues [137]. Studies indicate that tissue iron plays an important role in endurance training during submaximal exercise, with glucose being the main source of energy, whereas haemoglobin-bound iron plays a role in maximal aerobic exercise [5]. In the study by Gardner and Edgerton subjects with anaemia were found to transport 15% less oxygen per pulse and to restore their physical capacity with iron supplementation [138]. Iron supplementation also improves the resting metabolic rate (RMR). A study of two female athletes with ID supplemented with iron has shown an improvement in their thyrometabolic status and RMR at 8 and 16 weeks of the treatment [139].

## 8. Iron and Obesity

Around the world, 39% of adults are overweight and 13% are obese. The numbers have increased threefold since 1975. The most alarming figures are those about children and adolescents—nearly 380 million of them are overweight or obese. Obesity is not just a statistical problem, but it is the cause of numerous disorders including diabetes with its cardiovascular complications and chronic heart failure.

By inducing inflammation and hemodynamic and neurohormonal disorders, obesity may contribute to the development of chronic heart failure with preserved ejection fractions (HFpEFs) [140]. Obesity is also accompanied by sodium retention and abnormal cardiomyocyte metabolism of energy substrates [141]. Obesity and iron strongly interact with each other. The Western lifestyle is characterised by inappropriate eating habits, including eating in a hurry, and consuming junk foods-or fast foods of low nutritional value. Obese patients are more likely to develop iron deficiency. In the *NHANE-I* study Micozzi et al. [142] have found that a higher BMI was associated with lower iron and transferrin levels. Obese versus non-obese adults had lower iron levels, transferrin saturation, and MCV and significantly lower sTfR. In obese patients, ferritin and CRP levels were elevated and directly correlated with BMI [143]. Suggested hypotheses included increased blood volume in obese subjects [144] - chronic inflammation with subsequent changes in iron sequestration, and the effects of hepcidin and lipocalin-2, a protein with increased expression in white adipose tissue, serving as a siderophore binding protein (SBP), up-regulated in inflammation and impairing iron absorption [145]. Moreover, obesity affects the iron levels in the brain—mesencephalon and thalamus. In the midbrain, an increase in neurodegeneration markers (alpha-synuclein, F2-isoprastans) is observed [146]. Mitochondrial biogenesis is a key metabolic process for adipocyte differentiation. Knockdown of mitoferrin 1 and 2, the two proteins involved in iron storage in the mitochondria, results in a lower mitochondrial iron content in adipocytes, which by reducing mitochondrial oxygen use and intracellular ATP levels, leads to lower expression of adipogenic genes and reduced synthesis of lipids during adipocyte differentiation [147]. Interscapular brown adipose tissue (IBAT) contains even more mitochondria when compared with white adipose tissue, which is due to thermogenesis, with a key role played by iron [147]. In ID, an overgrowth of IBAT is seen with a paradoxical decrease in the effectiveness of thermogenesis [148]. A low temperature should lead to increased levels and turnover of thyroid hormones (accelerated transformation of T4 to T3 in the liver and IBAT and higher use of T3). These processes are blunted in ID [149]. Mitochondrial dysfunction in adipocytes may be involved in the development of type 2 diabetes [150]. An iron-rich diet resulted in body weight loss in obese mise with T2DM [151]. ID can also contribute to obesity because of fatigue, which is associated with lower physical activity [152]. Impairment of the mitochondrial respiratory chain reduces physical capacity and potentiates insulin resistance [153]. ID can lead to oxidative stress, which results in increased levels of proinflammatory cytokines, which in turn activate leucocytes and promote fat storage [154]. Other studies have shown that ID potentiates body weight increase and insulin resistance by reducing the activity of AMP-activated protein kinase [155].

Apart from white and brown adipose tissues, there is also bone marrow adipose tissue [156]. The tissue can have a negative impact on haematopoiesis through chemokine secretion and direct intercellular interactions [157]. Adipose cells and bone marrow cells originate from the same progenitor cells [158]. Excess adipocyte proliferation can lead to fatty degeneration of red bone marrow [156]. What is interesting is that a negative correlation is observed between the amount of bone marrow fat and haematopoiesis—when adipocyte mass increases, haematopoiesis decreases. Flozins inhibit intraorgan and visceral fat accumulation [159,160]. Dapagliflozin can reduce macrophage infiltration and improves lipid metabolism in murine bone marrow [161], as well as potentiates bone marrow haematopoiesis [162].

We must also consider the effects of bariatric surgery, which can also be the cause of iron deficiency. Studies show that within 10 years of the procedure ID develops in more than two-thirds of patients undergoing Roux-en-Y gastric bypass (RYGB) surgery in spite of the fact that a half of the studied patients takes iron supplements [163].

## 9. Iron and Diabetes

Diabetes affects 20–40% of patients diagnosed with heart failure [164]. HF patients with concomitant DM are at a significantly higher risk of morbidity while their risk of mortality is 8.8 times higher [165]. Insulin resistance (IR) occurs and develops as an inherent element of heart failure. Mechanisms underlying the pathogenesis of insulin resistance in heart failure are independent of the genetic and epigenetic susceptibility to diabetes [166]. IR is strongly associated with heart and skeletal muscle dysfunction [167]. IR correlates with NYHA class and peak oxygen consumption (peakVO2) [168]. Iron is an important regulator of glucose metabolism. The relationship between iron metabolism balance and diabetes is complex and has not been fully elucidated. A number of studies have shown a relationship between ferritin levels and risk of DM [169,170] and insulin resistance (IR) [171]. Irons blocks the insulin inhibitory effect on glucose production in the liver while insulin stimulates ferritin synthesis [172] and redistributes transferrin receptors to the cell surface [173]. Flemig has shown that numerous genes involved in iron metabolism (DMT1, ferroportin, MTP1) are changed in DM [174]. Muscle iron accumulation results in reduced glucose uptake [175], and increased pancreatic expression of DMT1 [176]. Instead, iron deficiency can increase insulin receptor expression and GLUT4 transcription in the muscles [177]. Some studies focus on iron overload-induced inflammation and generation of reactive oxygen species ROS), which can affect insulin resistance [178]. Another possible mechanism is the development of insulin resistance in brain iron overload [54,179]. As already mentioned, ID, by impairing mitochondrial respiratory chain, can reduce physical capacity and potentiate insulin resistance [153]. Although in iron deficiency anaemia (IDA) glucose and lactate levels are increased, their compensatory utilization due to improved insulin sensitivity is increased [136]. This process involves the modification of insulin receptor and changes in glucose transport. The study by Kemp et al. has confirmed an increase in gluconeogenesis in ID, in order to potentiate glucose delivery to tissues [137]. In the study by Wasserman et al. dogs with ID during exercise had higher levels of glucagon, cortisol, epinephrine and norepinephrine [135]. Ohira et al. have found higher blood glucose levels in animals with ID. Moreover higher activity of lactate dehydrogenase was seen in the muscles of animals with ID [136]. ID and IDA can contribute to more frequent diabetic complications and to poorer control of both type 1 and type 2 diabetes [180]. ID can increase the risk of diabetic nephropathy and retinopathy [181]. The incidence of type 2 diabetes mellitus was higher in the group of patients with chronic kidney disease and concomitant iron deficiency [182]. Ongoing studies investigate the effect of iron metabolism on diabetes via human microbiome modifications. The microbiome exerts its effect on iron absorption, which in turn modifies microbiome composition and metabolic activity, as well as gut immune system functionality [183]. A study has shown that the absence of beneficial gut microflora can impair iron absorption by as much as 25% [184]. In ID, cell sensitivity to bacterial endotoxins is increased [185]. As a result of intestinal bacterial fermentation, short chain fatty acids (SCFAs) are produced, which have the ability to modulate insulin sensitivity by affecting endotoxemia, inflammation and immune cascade activation. SCFAs bind to and activate G-protein coupled receptors (Gpr-1) on endocrine cells and regulate secretion of glucagon-like peptide 1 (GLP1) and peptide-YY (PYY) [186]. By binding to Gpr-1, endotoxins stimulate pancreatic beta-cell receptors and inhibit insulin secretion [187]. Moreover, microbiome stimulates bile acid secretion, whereas bile acids can impair insulin secretion [188]. However, caution is required while interpreting studies of pregnant women. Although some meta-analyses indicate that IDA can reduce the risk of gestational diabetes mellitus (GDM), there are also reports that show higher incidence of GDM in ID. Nevertheless the studies are too scarce to draw definite conclusions [189,190]. There are many other factors involved in the development of gestational diabetes, including body mass index (BMI) and the age of the mother [191]. Gestational diabetes can lead to the development of ID in the foetus. Newborn offspring of diabetic rat dams had a lower iron content in the heart, liver, and brain. Foetal hyperinsulinaemia leads to hypoxia, which results in increased EPO secretion and enhanced erythropoiesis, which in turn is responsible for increased iron requirement [192]. Studies in humans show that gestational diabetes results in iron deficiency in the foetus which is often accompanied by developmental disorders including memory deficits in the child [193]. ID in diabetic patients can increase the risk of cardiovascular disorders. It can cause dysfunction of the mitochondrial protein containing the Asn-Glu-Glu-Thr sentence (mitoNEET), which is an iron-containing protein located in the outer mitochondrial membrane and playing an important role in the regulation of maximal mitochondrial oxidative capacity [194]. This protein contains a redox-active pH-labile iron-sulphur, which can donate two iron and two sulphur atoms in chemical reaction. Therefore, MitoNEET functions as an iron-sulphur cluster, transferring the clusters in redox reactions [195]. ID impairs metabolic control in diabetes and increases the frequency of diabetic complications [181]. It is more common in patients with diabetic kidney disease [182], among whom patients with lower iron levels had elevated levels of inflammatory markers including hsCRP [196]. ID is also more common in patients with diabetic retinopathy, in whom it can accelerate the progress of macular oedema [197]. Finally, ID can substantially decrease the quality of life in patients who must cope with their disease for most of their lifetime—i.e., patients with type 1 diabetes mellitus [198]. Therefore, preventing and treating ID and IDA is beneficial in DM patients.

## 10. Iron and COVID-19

Infection with the new coronavirus SARS-CoV-2 can lead to heart failure in several mechanisms including direct cardiomyocyte damage, myocarditis, increased coagulability, and cytokine storm [199]. *Ferrum* is vital for key immune system reactions. TRF1 (a protein transporting iron into lymphocytes) gene mutation can cause considerable immune deficits in humans, with low IgG levels and reduced B and T cell proliferation [200]. Iron is necessary for normal activity of peroxidases and synthases involved in nitric oxide generation, which is of key importance for immune cell functionality. Iron is also involved in the regulation of cytokine production and affects signalling pathways [201]. In ID adequate cell response generation is delayed [202]. A number of studies in humans have confirmed innate immune response disorders in ID states. Macrophage bactericidal activity is impaired and neutrophil myeloperoxidase (MPO) producing reactive oxygen species responsible for intracellular pathogen death is reduced [203]. Children with ID were observed to have lower IgG and IL-6 levels and reduced phagocytosis and neutrophil oxidative burst response [204]. ID impairs T cell blastogenesis and mitogenesis as well as protein kinase C activity [205]. In mice, ID impairs T-cell mediated antigen-specific immunity and reduces antibody production and B-cell proliferation [206]. Moreover induction of cyclin S is reduced as well entry of B-cells into the S phase of the cell cycle [206]. Increased lymphocyte metabolism requires more iron while the T- and B-cell response to adenovirus and vaccinia virus is reduced in mice with ID [207]. The COVID-19 pandemic, which until now has killed 6 million 310 thousand people worldwide (as on 14 June 2022) is more dangerous for patients with ID [208]. Approximately 80% of those of who died from COVID-19 had functional iron deficiency owing to its sequestration, with elevated levels of IL-6 and hepcidin [209]. Studies have confirmed that functional ID was associated with longer duration of hospitalisation, five times higher risk of Intensive Care Unit admission and eight times higher risk of mechanical ventilation [209]. COVID-19, when replicating, interacts with host intracellular proteins, which are dependent on iron availability [210], including RNA reductase-therefore resistance to COVID-19 may directly depend on intracellular iron content. Lung function deterioration and hypoxia are the main negative consequences of COVID-19 and ID can exacerbate lung disease in response to low oxygen saturation [211,212] and alter macrophage polarization and cytokine production, which impairs the immune response to COVID-19 [89]. In rat studies ID led to increased production of hypoxia induced factor (HIF) 1 alpha, HIF2 alpha, nuclear factor of activated T cells (Nfat), survivining and signal transducing molecules promoting immune cell proliferation and resistance to apoptosis, which finally resulted in more rapid development of pulmonary vascular changes, pulmonary hypertension and heart failure [90]. In other studies loss of DMT1 expression in mice resulted in higher pulmonary susceptibility to bacterial infections and more rapid progression of lung tissue damage [213]. ID can alter the way patients experience the sensation of hypoxia [206] and as a result they may call for help when it is too late.

On 8 December 2020, 90-year old Margaret Keenan received the first ever dose of the COVID-19 vaccine. Until now complete vaccination against COVID-19 was administered to 4 billion 78 million people worldwide (as on 19 June 2022). Unfortunately the vaccination is not always effective. Apparently, the effectiveness of the vaccination can be altered by iron deficiency. The most recent evidence suggests impaired antigen-specific immunity as low iron levels in mice caused by hepcidin increase have been shown to reduce the immune response to the vaccination [207]. In a study with influenza virus ID reduced B and T cell count and decreased antibody production while iron supplementation improved the immune response to the vaccination [207]. This was confirmed in a study of 573 children from Kenya, which showed impaired immune response to trivalent poliovirus vaccine, diphtheria-tetanus vaccine, whole cell pertussis vaccine, Hemophilus influenzae vaccine, 10-valent pneumococcal vaccine and measles vaccine [199]. In this study, a group of children supplemented with iron at the time of their measles vaccination was found to have higher IgG antibody levels and higher avidity at 9 months after the vaccination when compared with children who were not supplemented with iron [214].

## 11. Iron and Enviromental Pollution

Technological progress and growing industrialization in developing countries have brought us to the point where the human body is exposed to thousands of chemical compounds coming from smog, the mining industry, agriculture, vehicle exhaust fumes or gas stove emissions. Groups of particles that exert biological effects include alcohols, diols, epoxides, ethers, aldehydes, ketones, hydrocarbons and ester groups. Exposure to pollutants may result in histopathological lesions caused by inflammatory infiltration, oedema and fibrosis. Moreover progression of inflammation and increased blood viscosity are observed [215,216]. The highest mortality and morbidity due to environmental pollution is caused by cardiovascular disorders [217]. Exposure to chemicals is associated with an increased risk of coronary artery disease and stroke [218]. The underlying mechanism involves endothelial dysfunction, which is of key importance for the arterial hypertension, diabetes mellitus, and atherosclerosis development [217]. In the MESA-Air study in a group of 6795 patients every 5 mcg/m^3^ increase in exposure to ambient particulate matter of 2.5 micrometres in diameter (PM 2.5) was associated with atherosclerosis progression, as assessed by coronary calcium score, by 4.1 Agatston units per year [219]. Iron homeostasis dysregulation can make the human body more sensitive to environmental pollutants. Through endocytosis functional groups of chemical molecules remove iron atoms from the inside of the cell, and cause its functional deficiency. Functional iron deficiency activates kinases and transcription factors that are able to trigger inflammation. Tissue damage occurs as a result of disrupted iron homeostasis following their exposure to chemical compounds. Increased availability of iron can reduce its cellular deficiency and eliminate the biological effects of chemicals [220]. Iron is kinetically preferred by chemical compounds because of its electropositivity, high affinity to oxygen-containing chemical groups and availability. Through endocytosis of inorganic particles their surface functional groups come into reaction with iron to produce chemical complexes [221]. In response to cellular iron loss activation of iron regulatory proteins (IRPs) occurs and iron import by TfR and divalent metal transporter 1 (DMT1) is increased. Chemical particles have the constant ability to export iron from cells while the body response to that process involves sequestration of this element to such an extent to make it unavailable to chemicals. Therefore iron is stored with ferritin and used for toxic ferrugionuous body formation [222]. Following vanadium exposure the amount of non-haem iron in the nucleus and mitochondria has rapidly decreased with its concomitant increase in cellular supernatant [223]. RNA for DMT1 increased four times after 4-h cell exposure to vanadium, which indicates an intensive iron import to the cell during the exposure. Vanadium is able to interact with iron bound to ferritin, transferrin and lactoferrin [224,225,226,227]. Cells administered iron prior to their exposure to pollutants demonstrated lower oxidant levels. Exposure to silica resulted in increased activation of NFkappaB and Nrf2-ARE pathways, which affected the expression of genes involved in inflammation. This led to increased levels of IL-6 and IL-8 although these cytokines were not released by cells rich in iron [226]. Studies in mice have shown that animals with ID exposed to cigarette smoke had higher levels of alveolar macrophages, higher levels of IL-6 and MCP-1 and were subject to more rapid changes in lung volume and functional residual capacity (FRC) [228]. ID resulted in higher brain sensitivity to manganese exposure [219]. Animals with ID presented more severe intracellular inflammation in response to smog exposure [220].

## 12. Iron and Stress

In the 17th century Thomas Willis suggested that emotions are born in the brain rather than in the heart, as believed in the past. The brain-controlled autonomic nervous system affects the heart and can cause Takotsubo syndrome (stress-induced cardiomyopathy), cardiac arrhythmia, progression of atherosclerosis, arterial hypertension and finally heart failure [229]. Stress is unavoidable in modern life but what is of key importance is the body’s hormonal response to it. Studies indicate that stress can damage the hippocampal structure through continued activation of the hypothalamic-pituitary-adrenal axis [230]. ID impairs the ability to maintain normal body temperature during exposure to cold [231] and during hypoxia. Plasma clearance of norepinephrine in ID states is considerably lower with impaired turnover of norepinephrine in tissues, and a concomitant increase in norepinephrine levels and decrease in thyroid hormone levels [232]. In ID a decrease in oxygen levels (down to 10 and 50% of baseline values) more rapidly caused hypothermia [233] and reduced oxygen transport to cells [234]. Moreover ID was found to impair cortisol secretion. Too low cortisol levels were observed after one hour of cold exposure [231]. In ID histological and ultrastructural changes occur in the adrenal glands. The most affected structures are mitochondria, which often become grossly enlarged and develop unusual electron-dense inclusions. Lipid droplets in iron deficient adrenal cells were much less developed compared with cells with a normal iron supply [235]. Impaired cortisol secretion is most likely caused by reduced activity of cytochrome P450 enzymes, omega-aminolevulinic acid synthase and ferrochelatase. Moreover, increased norepinephrine levels can suppress cortisol secretion [236]. In the study by Dallman et al. cortisol secretion was impaired in spite of increased ACTH levels and its response to stress was more blunted at night hours (although it should be more pronounced at night) [237]. Monkeys with ID presented abnormal cortisol secretion in the social stress test, in response to an intruder and in response to pictures presenting social and unsocial issues [238]. In a study of pregnant women higher cortisol levels were seen in ID versus control subjects [239]. Moreover subjects with iron deficiency are observed to have lower serotonin levels and reduced serotonin reuptake by synaptic vesicles as well as a blunted behavioural response to SSRI administration [240]. Stress also increases the production of prolactin. Iron deficiency affects prolactin levels as prolactin is a peripheral marker of central dopamine release. Dopamine released from tuberoinfundibular dopaminergic neurons (TIDA) inhibits the release of prolactin from the anterior pituitary gland [240]. Studies have shown that early maternal care of offspring was associated with lower prolactin levels in later life in response to stress [241]. The most recent research shows that children born to mothers with ID had higher prolactin levels in the umbilical cord blood [242]. High prolactin levels in foetal life and childhood may set a new balance in TIDA neurons and subsequently counteract the beneficial effect of maternal care on prolactin levels and increase the offspring sensitivity to stress in later life.

## 13. Iron and Psychiatric Disorders

Alterations in gene expression and neurotransmitter metabolism in ID can lead to neuropsychiatric disorders [243,244]. Depression is reported in 20% of HF patients (from 11% of NYHA I up to 42% of NYHA IV patients) and is an independent risk factor of mortality and reduced QoL in the group of patients with HF [245]. Depressive anxiety disorders strongly interact with health failure in a vicious circle of declining health outcomes. Depressive episodes are more common following HF exacerbations. In a rat study, a 65% reduction in willingness to explore new environment was found, which correlated with iron levels in the midbrain and DA1 receptor density in the midbrain and caudate nucleus [246]. A 45% variability in latency and higher willingness to move to a safer environment, which may correspond to the development of anxiety disorders in human, is associated with the number of DA transporters and density of D2 receptors in the nucleus accumbens [247]. ID in rats is associated with a lower willingness to explore new environments [246]. Prenatal ID results in an increased frequency of anxiety behaviours in young rats, which is only partially reversible upon iron supplementation [248]. The prevalence of depression is higher in the group of subjects with ID [249], and children born to ID mothers are at four times higher risk of schizophrenia spectrum disorders versus controls. Every 1 g/dL increase in maternal Hb level resulted in 27% decrease in the risk of SDD [250]. ID contributes to lower IQ and potentiates symptoms of ADHD in the group of adopted children [251], with lower iron levels in the thalamus [252]. ID impairs brain performance-as shown by EEG results, children with iron deficiency had slower activation of potentials [253]. An amount of 50% of HF patients complain of sleep disorders [254], and the disorders are even more severe in patients with acute heart failure [255]. While in the 21st century effective use of every single hour of rest is extremely important, people with ID have a reduced sleep spindle density, a decreased frequency and increased duration of inter-spindle intervals during non-rapid eye movement (NREM) sleep and slow wave sleep (SWS) [256].

## 14. Conclusions

Aging of the societies of developed countries in the 21st century has lead to the predominance of cardiovascular disorders. However, promising new treatment options provide hope for better outcomes in patients with chronic diseases, such as heart failure. Considering the technological and medical progress of the 21st century, we should always bear in mind migrations, epidemics, hunger and malnutrition in still developing countries. Iron deficiency is global issue that potentiates cells sensitivity to heavy metals and chemical particles, which may lead to a range of cardiovascular disorders. It also impairs the body’s reactions to stress, which is unavoidable in our life, leading to sympathetic nervous system hyperactivity and accelerating the progress of atherosclerosis. Finally, it can impair the immune response to viral infections, including COVID-19, and reduce the effectiveness of vaccinations. We are facing the risk of new pandemics, with one of the potential threats being the monkeypox virus, which already is a global problem spreading not only in Africa but also in other regions of the world. Having to face numerous challenges of the current world, active surveillance of iron deficiency seems to be of high significance from the public health point of view as this element possibly plays a crucial role not only in the terms of widely understood cardiovascular health.

## Figures and Tables

**Table 1:** A Interventional studies of iron deficiency in heart failure.

Study Name/Author	Iagnosis	Number of Patients	Mean Age	Ferritin, Tsat and Hb	Exclusions Criteria	Nyha	Lvef	Nt-Probnp	Dosage of Iron	Duration	Effect on Hematologic Parameters	Results/Primary and Secondary End Points	Safety
Bolger et al. [85]	HFrEF	16	68.3 ± 11.5	Hb < 12 g/dL; ferritin <30 ng/mL; transerrin saturation <16%	Vitamin B1, Folic acid deficiency; hemoglobinopathy; ferritin > 400 ng/mL	NYHA II–III	<26 ± 13%	Not included	Iron sucrose 200 mg on days 1, 3 and 5. If ferritin < 400 ng/mL on day 12–200 mg doses on days 15 and 17	12 weeks	Hb 11.2 --> 12.6 g/dL; ferritin 87 --> 217 ng/mL; TSAT 16 --> 24.6%	Increased 6MW MLHF from 33 --> 19;	Well tolerated
Toblli et al. [86]	HFrEF	40	A—intervetion group: 74 ± 8; B—control group: 76 ± 7	Hb < 12,5 g/dL; ferritin < 100 ng/mL; TSAT <20%	Patients with: hemodialysis therapy; NYHA I; allergy to iron; acute bacterial infections; neoplasm; parasitism; chronic digestive disease; hypothyroidism; congenital cardiopathies; receiving iron or rhEPO in the previous 4 weeks; history of hospitalization during previous 4 weeks	NYHA II–IV	<35%	Included	Iron sucrose 200 mg weekly for 5 weeks	25 weeks	Hb 10.3 --> 11.8 g/dL; ferritin 78.9 --> 240.4 ng/mL; TSAT: 20 --> 25%	In group B: nt-proBNP: 450.9 --> 117.5 pg/mL, CRP: 6.5 --> 2.3 mg/mL; LVEF: 28.8 --> 35.7%; NYHA 3.3 --> 2.0; MLHFQ: 59 --> 41; 6MW test 184.5 m --> 240.1 m	–
FAIR-HF/Anker et al. [88]	HFrEF	459	-	Ferritin < 100 ng/mL; or Ferritin 100–299 ng/mL with TSAT <20%; Hb: 9–13.5 g/dL	Uncontrolled hipertension; Inflammation; impaired liver or renal function	NYHA II–III	<40% in NYHA I; <45% in NYHA III	Not included	200 mg ferric carboxy- maltose	53 weeks	The mean difference in the ferritin level between group receiving iron and those receiving placebo was: 246 ± 20 mcg/L at week 24. Hb: 5.9 ± 1.5 g/L;	Patient Global Assessment in the group receiving iron was improved with 50% of patients as compared to 28% in the pla- cebo group. 47% having NYHA I–II in the group receiving iron, as compared with 30% in the placebo group. Improvement in QoL and 6MW test in the gruop receiving iron.	No severe allergic reactions. Injection discoloration in 4 and injection-site pain in 2 patients.
FERRIC-HF [89]	HFrEF	35	-	Ferritin < 100 ng/mL, or ferritin < 100–200 ng/mL with TSAT <20%; Hb < 12 g/dL anemic group	Use of Epo oraz iron; blood transfusion within the previous 30 days; history of hemochromatosis (or first relative with hemochromatosis); hypersensitivity to parental iron; history of allergic disorders; active infection, bleeding, malignancy or hemolytic anemia; decompensated heart failure; muscosceletal disease; unstable angina pectoris; obstructive cardiomyopa- thy; severe uncorrected valvular disease; uncontrolled brady- or tachyarrhytmias; immunosuppressive or renal replacement therapy; chronic liver disease	NYHA II–III	<45%	Not included	Iron sucrose 200 mg weekly until ferritin >500 ng/mL	16 weeks	Ferritin: 151 --> 396 ng/mL; TSAT 5% --> 17%; sTfR 1.4 --> 1.3 mg/L; Hb 8 g/dL --> 9 g/dL	NYHA: 2.4 ± 0.5 --> 2.1 ± 0.5; MLHFQ score: 41 ± 22 --> 31 ± 25; Fatigue score: 6 ± 1 --> 4 ± 2; LVEF: 30 ± 7 --> 32 ± 10; Absolute pV02: 13.9 ± 2.7 --> 15.4 ± 3.5 mL/kg/min	No episodes of symp- tomatic hypotension oraz anaphylactic reactions
CONFIRM-HF [90]	HFrEF	304	–	Ferritin < 100 ng/mL, or ferritin 100–299 ng/mL with TSAT <20%; Hb <15 g/dL	Uncontrolled hipertension; infection; malignancy; impaired liver or renal function	NYHA II–III	<45%	BNP > 100 pg/mL; NT-proBNP >400 pg/mL	200 mg ferric carboxy- maltose	52 weeks	Mean treatment effect on ferritin and TSAT in patients assigned to ferric carboxy- maltose compared to placebo was 265 ± 19 ng/mL and 8.9 ± 1.1% at week 24 and 200 ± 19 ng/mL and 5.7 ± 1.2% at 52 week. Hb: 0.6 ± 0.2 and 1.0 ± 0.2 g/dL, at weeks 24 and 52, respectively.	Increase in 6MWT distance by 18 ± 8 m in ferric carboxymal- tose group. Decrease in 6MWT distance by 16 ± 8 m in placebo group. Significant benefit in PGA and NYHA. Reduction in Fatigue score. Beneficial effect on QoL (overall KCCQ) and EQ-5D health state score.	No severe allergic reactions. Two patients experienced injection site discolouration, four patients repor- ted feeling hot. One patient reported urticaria, rash and erythema.
EFFECT-HF [91]	HFrEF	174	–	Ferritin < 100 ng/mL, or ferritin 100–299 ng/mL with TSAT <20%; Hb <15 g/dL	Known sensivity to ferric carboxymaltose; history of iron overload; received erythropoiesis-stimulating agents, i.v. iron therapy and/or blood transfusions in the 6 weeks before randomization.	NYHA II–III	<45%	BNP >100 pg/mL; NT- -proBNP >400 pg/mL	500 mg ferric carboxy- maltose 3 times	24 weeks	Hb: 12.9 ± 1.3 g/dL --> 13.9 ± 1.3 g/dL; ferritin: 48 --> 283 ± 150 ng/mL; TSAT: 17.3% --> 27.8 ± 8%	Increase peak VO2: + 0.25 mL/min/kg for ferric carboxymaltose versus −0.34 mL/kg/min for the usual care patients (difference: 0.45 ± 0.38). 11 patients hospi- talized, no deaths occured for HF in ferric carboxymaltose group. In the standard care group: 4 patients died during the study, 5 patients were hospita- lized for worsening HF	No hypersensivity reactions, no cases of hypophosphatemia
FERRIC-HF II [94]	HFrEF	40	–	Ferritin < 100 mcg/L, or 100–300 mcg/L with TSAT < 20%	History of acquired iron overload; known hemochromatosis or first degree relatives with hemachromatosis; an allergic disor- der; prior hypersensivity to iron drugs; active infection, bleeding, malignancy, hemolytic anemia, rheumatoid arthritis and myelodysplasia; AIDS; chronić liver disease; chronic lung disease; coagulopa- thy or anticoagulated for metallic valve or LV thrombus; immunosupressant use; renal dialysis; need for Epo or blood trans- fusions; unstable ungina; severe obstruc- tive lesions; uncontrolled arrhythmias; muscoloskeletal limitations	NYHA II–III	<45% if NYHA III; <40% if NYHA II	–	Iron isomaltoside 1000. The total repletion dose was calculated to the nearest multiple of 100 mg using the Ganzi formula: body weight (kg) × 2.4 × (15-patients Hb (g/dL) + 500 mg). Mean dose was 929 mg ± 320 mg	27 weeks	Ferritin increased by 83% (327 ± 185 ng/mL) in the iron group and decreased by 24% in the placebo group. TSAT incresed by 29% (8 ± 6%) with iron and by 4% (2 ± 9%) with placebo. Hb increased by 0.4% (0.6 ± 9 g/dL)	PCr t1/2 improved (shortened) by 17% in the iron group, worse- ned by 7% in the placebo. Improved post-exercise ADP t1/2 by 45% while it leng- thened by 3% with placebo. Reduction in NYHA and post-exercise Borg dyspnea score in iron group.	1 patient had arthralgia during the infusion; 1 noted a mild rash at the venepuncture site 1 day post-infusion; 1 had a serious adverse event-unrelated to stu- dy drug. No anaphy- lactic reactions.
RON-HF [82]	HF	23	–	Ferritin < 500 ng/mL; TSAT: <20%; Hb: 9–12 g/dL	Overt bleeding; hypothyroidism; inflam- matory, neoplastic or infectious disease; serum creatinine>1.5 mg/dL; intolerance to iron; HF due alcoholic cardiomyopathy; current regular drinker of alcohol; decom- pensated HF; recent ACS, stroke or TIA; recent myocardial revascularization pro- cedures; patients in heart transplantation list; pregnant or lactating women; pre- -menopausal women that are not using any method of contraception; patients with pacemakers, implanted defibrillators or cardiac resynchronization therapy	NYHA II–IV	<40%	Not included	Group 1: iron sucrose 200 mg i.v. once a week, for 5 weeks; group 2: ferrous sulfate 200 mg orally ×3/day for 8 weeks;	8 weeks	Ferritin increase in i.v. iron (167 ± 149 ng/mL to 293 ± 270 ng/mL) as well as in oral iron group (115 ± 141 ng/mL to 218 ± 189 ng/mL). No ferritin increment in pla- cebo group. TSAT increased in all study groups.	>20% improvement in maxi- mal oxygen consumption (V02 max) in i.v. iron from baseline. 4.36 mL/kg/min in V02max between i.v. iron and oral iron groups. 3.5 mL/kg/min intra- group increment detected in the i.v. iron group.	No data

## Data Availability

No new data were created or analyzed in this study. Data sharing is not applicable to this article.
